# *Lactobacillus plantae* Expressing Porcine Reproductive and Respiratory Syndrome Virus (PRRSV) Single-Chain Antibody Can Inhibit PRRSV Replication and Change the Intestinal Flora Structure of Piglets

**DOI:** 10.3390/ijms26052257

**Published:** 2025-03-03

**Authors:** Tianming Niu, Tianqi Fan, Yingjie Wang, Kuipeng Gao, Jinhui Zhao, Ruyu Wang, Xiaolei Chen, Junhong Xing, Jingjing Qiu, Boshi Zou, Shuhui Fan, Shi Zhang, Qiong Wu, Guilian Yang, Nan Wang, Yan Zeng, Xin Cao, Yanlong Jiang, Jianzhong Wang, Haibin Huang, Wentao Yang, Chunwei Shi, Zhipeng Li, Chunfeng Wang

**Affiliations:** 1 College of Veterinary Medicine, Jilin Agricultural University, Changchun 130012, China; niutianming@jlau.edu.com (T.N.);; 2 Jilin Provincial Engineering Research Center of Animal Probiotics, Jilin Provincial Key Laboratory of Animal Microecology and Healthy Breeding, Jilin Agricultural University, Changchun 130000, China; 3 Key Laboratory of Animal Production and Product Quality Safety of Ministry of Education, Jilin Agricultural University, Changchun 130000, China; 4 College of Animal Science and Technology, Jilin Agricultural University, Changchun 130000, China

**Keywords:** single antibody, PRRSV, recombinant lactobacillus, vaccine

## Abstract

Porcine reproductive and respiratory syndrome (PRRS) is an infectious disease that can cause reproductive disorders in sows and affect the breathing of piglets, seriously endangering pig breeding worldwide. In this study, *Lactobacillus plantarum* NC8 was used as the expression delivery vector of foreign proteins, and a single-chain antibody was designed based on an mAb-PN9cx3 sequence. Three recombinant strains of *Lactobacillus plantarum*, namely, NC8/pSIP409-pgsA‘-PN9cx3-scFV(E), NC8/pSIP409-pgsA’-PN9cx3-HC(E), and NC8/pSIP409-pgsA‘-PN9cx3-LC(E), were successfully constructed. In an in vitro test, the viral load of each experimental group was significantly lower than that of the control group (*p* < 0.01). In the piglet challenge protection test, the percentage of CD3^+^CD8^+^T cells in the blood of piglets given complex lactic acid bacteria was significantly increased before and after the challenge (*p* < 0.01); the body temperature of piglets in this group was normal, the viral load of each organ was reduced, and the obvious pathological changes in each tissue were alleviated. At the same time, the abundance of Bacteroides, Fusobacterium, and other bacteria in the intestinal tracts of the piglets changed, affecting the metabolism of carbohydrates and amino acids and the differentiation of Th1 and Th2 cells. This experiment provides a feasible strategy and method for the design of a PRRSV vaccine.

## 1. Introduction

Porcine reproductive and respiratory syndrome (PRRS), commonly known as “Blue-ear disease of swine” in China, is a positive-strand RNA virus with easy mutation [1]. Domestic pigs and wild boars are known hosts of infection. Sow infection can cause reproductive disorders [2]. It is an important pathogen that has a great impact on the pig industry worldwide. Small molecular antibodies can be highly expressed in bacteria with small molecular weight, strong penetration, and high specificity [3]. They are widely used in the prevention and detection of diseases and are expected to become a new way to replace traditional treatment forms [4]. PRRSV ORF3 encodes a very important GP3 envelope protein, which has strong antigenicity and virus clearance [5]. A certain level of antibodies against the GP3 protein could be detected in a host invaded by PRRSV [6]. Some experts believe that when GP3 protein neutralizes virus pathogenicity [7], virus infections may be related to M protein synergism.

Lactobacillus is a bacterium that can forage carbohydrates and produce large amounts of lactic acid [8]. It colonizes the human or animal gut over a long period and has many benefits for the body [9]. Lactobacillus bacteria are internationally recognized as safe microorganisms of food-grade [10]. Lactobacillus has the advantages of easy cultivation, simple operation, and high safety and has become the best choice for live vector vaccines expressing heterologous proteins and antigens in the field of genetic engineering vaccines [8]. Previously, our laboratory constructed many recombinant lactic acid bacteria vaccines for the prevention of pig diseases, and all of them have good immune effects [11]: Yang Wentao et al. used *Lactobacillus plantarum* to anchor the expression of a TGEV antigen (S) on *Lactobacillus plantarum* with a dendritic cell-targeting peptide (DCpep) [12]. Jin Yubei et al. constructed a set of recombinant *Lactobacillus plantarum* strains exhibiting spike proteins from TGEV and fused them with a DC-targeting peptide [8].

In this study, three strains expressing an anti-GP3 protein single-chain antibody, namely NC8/pSIP409-pgsA’ -PN9cx3-scFV(E), NC8/pSIP409-pgsA’ -PN9cx3-HC(E), and NC8/pSIP409-pgsA’, were constructed, creating a recombinant strain of *Lactobacillus plantarum*, PN9cx3-LC(E). According to the literature, the effect of compound lactic acid bacteria is higher than that of a single preparation. Therefore, three strains of recombinant lactic acid bacteria were combined to immunize piglets. The percentage of CD3^+^CD8^+^T cells in the blood of the piglets in the compound lactic acid bacteria group was significantly increased before and after the challenge, the viral load of all organs was significantly decreased, and the pathological changes in all tissues were significantly alleviated. The compound lactic acid bacteria have the potential to protect piglets from PRRSV challenges, providing a new strategy for preparing an oral lactic acid bacteria vaccine for porcine reproductive and respiratory syndrome.

## 2. Results

### 2.1. Lactobacillus plantarum Anchors and Expresses PRRSV Single-Chain Antibody and Prokaryotic Expression

**Figure 1:**
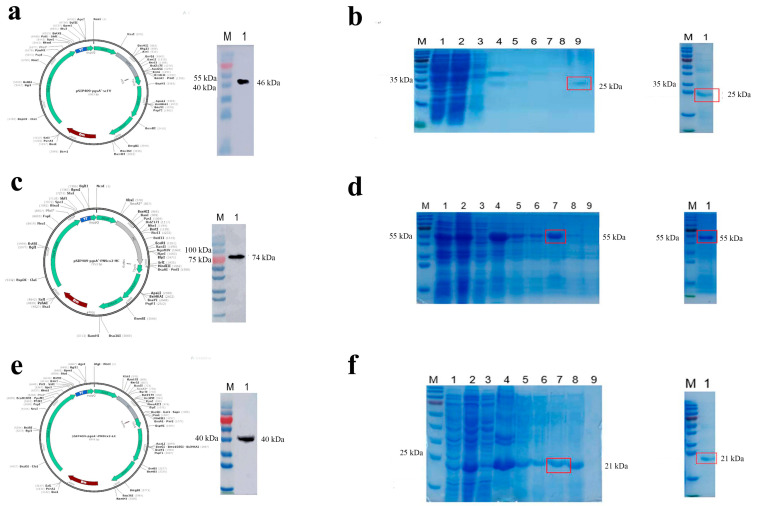
Construction diagram of Lactobacillus vector expressing PRRSV single-chain antibody and single-chain antibody protein purification results: (**a**) construction of pSIP409-pgsA ‘-SCFV carrier; (**b**) pET28a-PN9cx3-HC (BL21) PAGE results and protein purification; (**c**) construction of pSIP409-pgsA ‘-PN9cx3-HC vector; (**d**) pET28a-PN9cx3-scFV (BL21) PAGE results and protein purification; (**e**) construction of pSIP409-pgsA ‘-PN9cx3-LC vector; (**f**) pET28a-PN9cx3-LC (BL21) PAGE results and protein purification.

Three recombinant plasmids, pSIP409-pgsA ‘-PN9cx3-scFV (E), pSIP409-pgsA’ -PN9cx3-HC (E), and pSIP409-pgsA ‘-PN9cx3-LC(E), expressing anti-PRRSV GP3 scFV were successfully constructed (Figure 1a,c,e). Three recombinant plasmids were transformed into *Lactobacillus plantarum* NC8 via electroporation, and the proteins were obtained using the induction and repeated freeze–thaw method for SDS and Western blotting. After incubation with a His-tag mouse monoclonal antibody, the protein bands of 46 KDa (PN9cx3-scFV), 74 KDa (PN9cx3-HC), and 40 KDa (PN9cx3-LC), which matched the expected sizes, were observed (Figure 1a–c). Three recombinant plasmids expressing PRRSV GP3 scFV (E), namely, pSIP409-pgsA ‘-PN9cx3-scFV (E), pSIP409-pgsA’ -PN9cx3-HC (E), and pSIP409-pgsA ‘-PN9cx3-LC(E), were successfully expressed in L. plantarum. The pET28a-PN9cx3-scFV, pET28a-PN9cx3-HC, and pET28a-PN9cx3-LC plasmids were successfully transformed into BL21(DE3), induced by IPTG, processed into protein samples, and detected via SDS-PAGE. The results showed that there were clear protein bands at 25 kDa, 55 kDa, and 21 kDa, respectively, and the bands were in accordance with the expected size (Supplementary Figure S1). The three groups of target proteins were induced and purified for SDS-PAGE detection. The results showed that there were clear protein bands at 25 kDa, 55 kDa, and 21 kDa, which were in line with the expected band sizes (Figure 1b,d,f), indicating that the proteins of the three PRRSV scFV were successfully purified.

### 2.2. Antiviral Effect in Vitro

**Figure 2:**
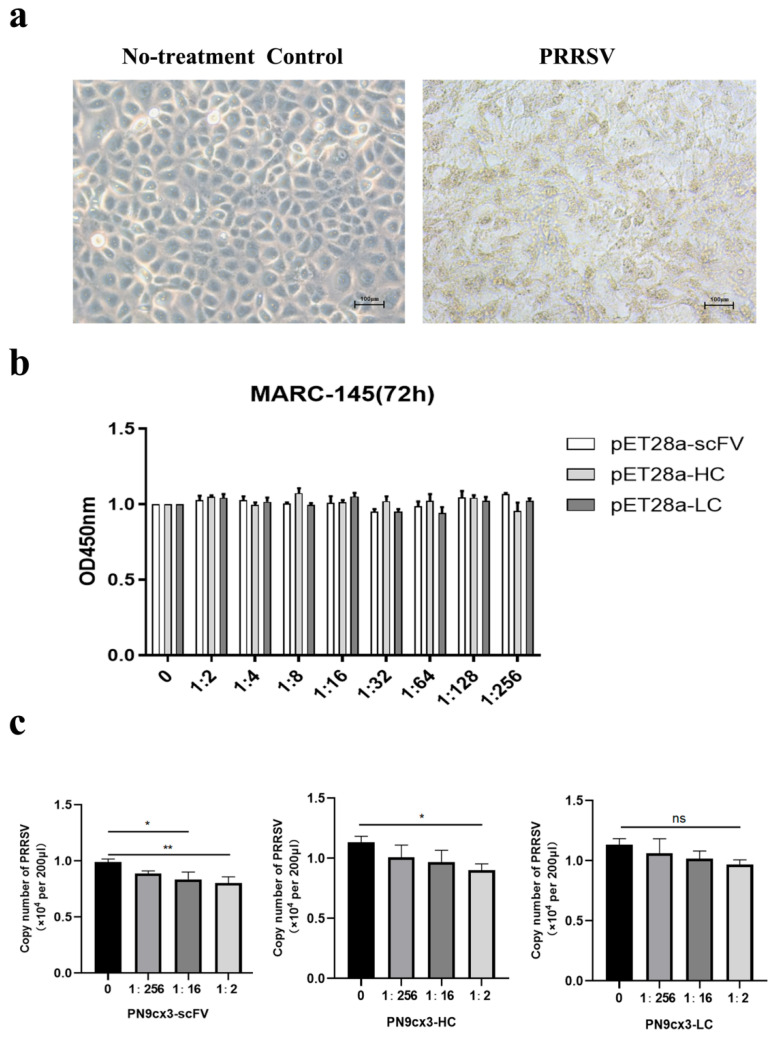
In vitro cell assay: (**a**) PRRSV infects MARC-145 cells; (**b**) interaction of different concentrations of single-chain antibody protein with MARC-145 cells; (**c**) inhibition of PRRSV in MARC-145 cells with different concentrations of single-chain antibody protein (*, *p* < 0.05; **, *p* < 0.01; ns, *p* > 0.05).

For MARC-145 cells, the cytotoxicity test of three groups of proteins against MARC-145 was started, and the results showed that MARC-145 had no obvious cytotoxicity under the action of different concentrations of proteins (Figure 2a,b). In this experiment, different concentrations of PN9cx3-scFV, PN9cx3-HC, and PN9cx3-HC proteins were used for neutralization tests with PRRSV and MARC-145 cells. The results were as follows: The PN9cx3-scFV protein was significantly different from the control group at a concentration of 1:2 (*p* < 0.01), and it was also significantly different at a concentration of 1:16 (*p* < 0.05). It was shown to have a neutralizing effect against PRRSV in MARC-145 cells (Figure 2c). The PN9cx3-HC protein was significantly different from the control group at a 1:2 concentration (*p* < 0.05) (Figure 2c). The results showed that both PN9cx3-scFV and PN9cx3-HC could neutralize PRRSV, while PN9cx3-LC was not significantly different from the control group at different concentrations.

### 2.3. Changes in Clinical Characterization of Experimental Pigs After Challenge

**Figure 3:**
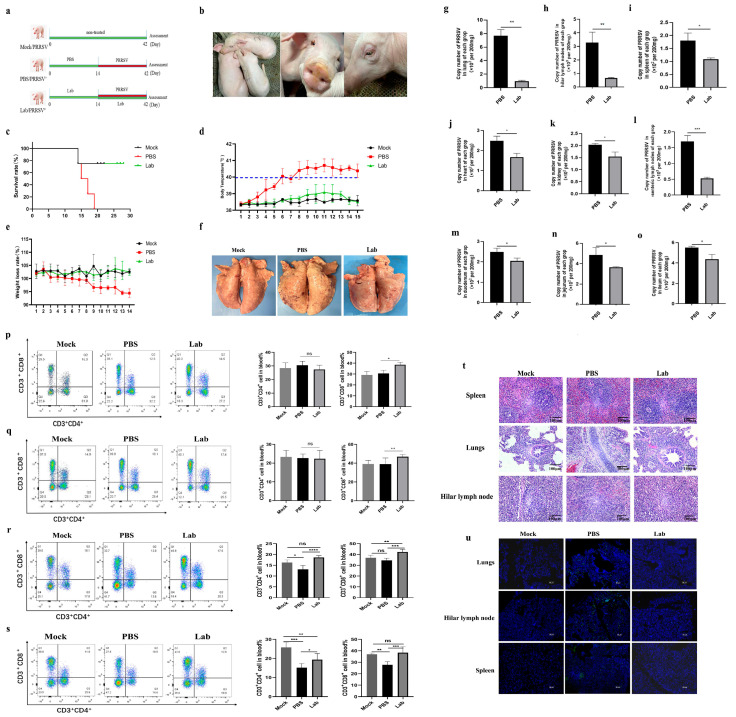
Challenge protection test: (**a**) grouping in animal experiments; (**b**) mental state of piglets infected with PRRSV; (**c**) survival curve; (**d**) temperature change; (**e**) weight change; (**f**) general observation of lungs of piglets in each group; (**g**) comparison of viral load in lungs of piglets in each group; (**h**) comparison of viral load in hilar lymph nodes of piglets in each group; (**i**) comparison of viral load in spleen of piglets in each group; (**j**) comparison of viral load in heart of piglets in each group; (**k**) comparison of viral load in kidneys of piglets in each group; (**l**) comparison of viral load in mesenteric lymph nodes of piglets in each group; (**m**) comparison of viral load in duodenum of piglets in each group; (**n**) comparison of viral load in jejunum of piglets in each group; (**o**) comparison of viral load in ileum of piglets in each group (*, *p* < 0.05; **, *p* < 0.01; ***, *p* < 0.001; ns, *p >* 0.05); (**p**) on the 7th day after immunization, T lymphocytes in peripheral blood of piglets in each group were detected with flow cytometry; (**q**) on the 14th day after immunization, T lymphocytes in peripheral blood of piglets in each group were detected with flow cytometry; (**r**) on the 5th day after challenge, T lymphocytes in peripheral blood of piglets in each group were detected with flow cytometry; (**s**) on the 10th day after challenge, T lymphocytes in peripheral blood of piglets in each group were detected with flow cytometry (*, *p* < 0.05; **, *p* < 0.01; ***, *p* < 0.001; ****, *p* < 0.0001); (**t**) results of histopathological examination of piglets in each group; (**u**) results of tissue immunofluorescence of piglets in each group.

In the animal experiment, the body temperature, body weight, and clinical characteristics of piglets in each group were monitored for the duration of the challenge. The Mock group had clinical symptoms at 3 dpi: drowsiness, respiratory distress, and a sudden rise in body temperature to 40 °C. At 4 dpi, the piglets in the Lab group developed asthma and other symptoms, and their body temperature was higher than in the Mock group. At 6 dpi, the body temperature of piglets in the Mock group reached 40 °C, and typical symptoms such as a loss of appetite, depression, runny nose, and aggravation of respiratory problems occurred. The piglets in the Mock group died at 15 dpi, 17 dpi, and 19 dpi (Figure 3c). From 7 dpi, the piglets showed a loss of appetite, lethargy, eyelid edema, and high fever, and the highest body temperature reached 41.1 °C (Figure 3d). The body temperature of the piglets at 12 dpi was still higher than 40 ° C, and no obvious symptoms were observed in the Lab group (Figure 3d). In the Mock group, loss of appetite due to infection was manifested as gradual weight loss, while in the Lab group, weight gradually increased (Figure 3e).

### 2.4. Poison Attack Pigs’ Autopsy Results After the Experiment

Fourteen days after PRRSV-JXA1 infection, one piglet from each group was sacrificed for anatomical observation, and the pathological changes in each organ were photographed. In the Mock group, we found multiple bleeding spots in the lungs, with obvious edema and lobular consolidation (Figure 3f). There was bleeding in the intestine, whereas no significant lesions were seen in the Lab group.

### 2.5. Virus Quantification Results of Each Tissue and Organ

Viral nucleic acids were extracted from the lung, hilar lymph nodes, spleen, heart, kidney, mesenteric lymph nodes, duodenum, jejunum, and ileum tissues of the piglets in the Lab group and Mock group after the challenge, and the virus was quantitatively analyzed by means of reverse transcription and qRT-PCR. The results showed that the viral load of lung samples in the Mock group was higher than in the Lab group (*p* < 0.01) (Figure 3g). The viral load of lung lymph node samples in the Mock group was higher than in the Lab group (*p* < 0.01) (Figure 3h). The viral loads of spleen, heart, kidney, mesenteric lymph node, duodenum, jejunum, and ileum samples in the Mock group were significantly higher than in the Lab group (*p* < 0.05) (Figure 3i–o).

### 2.6. Results of Viral Load Determination in Serum After Challenge

**Figure 4:**
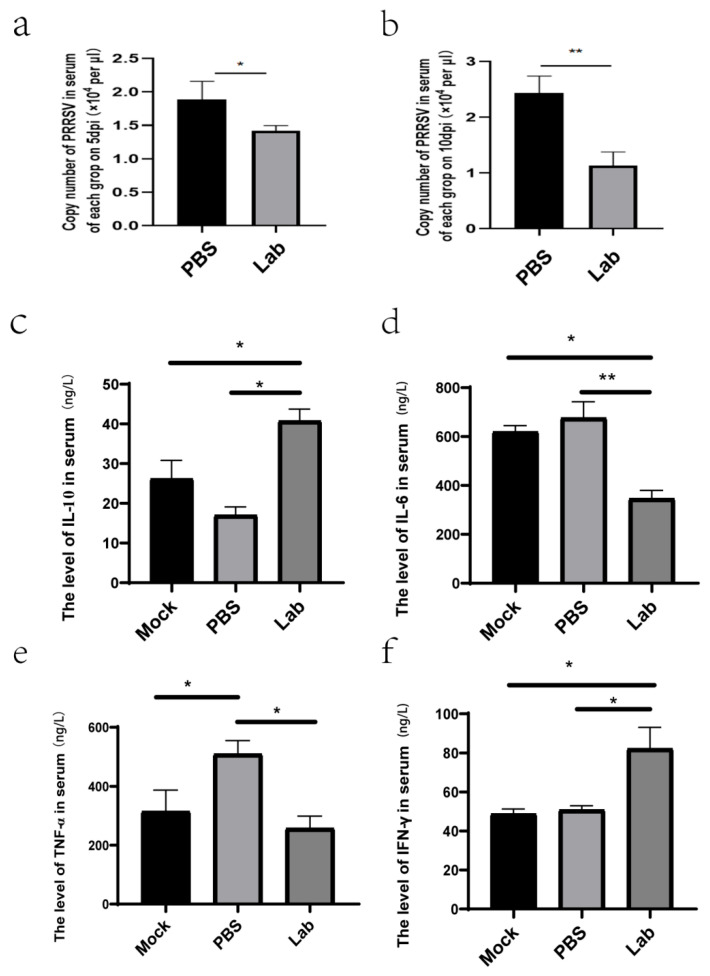
Quantitative analysis of viral vectors and cytokine detection in serum at different dates: (**a**) comparison of viral load in serum of piglets in each group on day 5; (**b**) comparison of viral load in serum of piglets in each group; (**c**) changes in IL-10 in serum; (**d**) changes in IL-6 in serum; (**e**) changes in TNF-α in serum; (**f**) changes in IFN-γ in serum(*, *p* < 0.05; **, *p* < 0.01).

The virus nucleic acid was extracted from the serum of the piglets in the Lab group and Mock group on the 5th and 10th day after the challenge and reverse-transcribed into cDNA. The virus was quantified via qRT-PCR. The results showed that the viral load of the serum samples in the Mock group reached 2.2 × 10^4^ copies/μL at 5 dpi. The serum viral load in the Lab group was significantly lower (*p* < 0.05) than in the Mock group (Figure 4a). At 10 dpi, the serum viral load in the Lab group decreased to 1.4 × 10^4^ copies/μL, and the serum viral load in the Lab group was significantly (*p* < 0.01) lower than in the Mock group (Figure 4b).

### 2.7. ELISA Analysis

Serum cytokines IL-10, IL-6, TNF-α, and IFN-γ were detected. The results showed that compared with the Mock group, IL-10 in the Lab group was significantly increased, while in the PBS group, it was significantly decreased (*p* < 0.01) (Figure 4c). IL-6 in serum was significantly decreased in the Lab group compared with the Mock group, and there was a difference between the PBS and Lab groups (*p* < 0.05) (Figure 4d). There were significant differences in serum TNF-α between the PBS group and the Mock and Lab groups (*p* < 0.01) (Figure 4e). Serum IFN-γ was significantly higher in the Lab group compared to the Mock group (*p* < 0.01) (Figure 4f). Lactic acid bacteria have good anti-inflammatory and antiviral effects on piglets.

### 2.8. Localization of Virus in Tissues via Immunofluorescence

Immunofluorescence was used to detect virus infections in the heart, spleen, lungs, kidneys, hilar lymph nodes, mesenteric lymph nodes, duodenum, jejunum, and colon. The results showed that the fluorescence intensity of the virus in the lungs, hilar lymph nodes, and spleen of the piglets in the Mock group was higher than in the Lab group at the late stage of infection (Figure 3u). The viral fluorescence intensity of the heart, kidney, mesenteric lymph node, duodenum, jejunum, and ileum tissue of the piglets in the Mock group was higher than in the Lab group (Supplementary Figure S1). According to the results of immunofluorescence, PRRSV caused varying degrees of damage to the piglets after infection in each group, and pathological particles in the heart, kidneys, spleen, hilar lymph node, mesenteric lymph node, duodenum, jejunum, and colon of piglets orally treated with lactic acid bacteria were significantly reduced, indicating that lactic acid bacteria had a good protective effect on piglets.

### 2.9. Histopathological Observation

Histopathological observations of all groups showed significant pulmonary edema and interstitial pneumonia in the Mock group (Figure 3f). There was also splenic red blood cell infiltration, organ bleeding, pulmonary alveolar cell structure destruction, and thickened alveolar walls, which were full of inflammatory exudates and inflammatory cells, and there was also a large number of inflammatory exudates and a large amount of immune cell infiltration in the hilar lymph nodes (Figure 3t). Myocardial cell injury in the Mock group could lead to myocarditis, and there were hemorrhagic spots in the duodenum, jejunum, and ileum, while there were no obvious pathological injuries in the Lab group and Mock group (Supplementary Figure S2).

### 2.10. Flow Cytometry of Peripheral Blood T Lymphocytes After Challenge

On the 7th and 14th days of immunization, the flow detection results of peripheral blood T lymphocytes in the PBS and Lab groups showed that on the 7th day of immunization, the CD3^+^CD8^+^T cells in the Lab group were significantly higher than those in the PBS group (*p* < 0.05). There was no significant difference in the proportion of CD3^+^CD4^+^T cells between the Lab group and the PBS group (Figure 3p). On day 14 after immunization, the proportion of CD3^+^CD8^+^T cells in the Lab group was significantly higher than in the PBS group (*p* < 0.01). There was also no significant difference in the proportion of CD3^+^CD4^+^T cells between the Lab group and the PBS group (Figure 3q).

The flow cytometry results of peripheral blood T lymphocytes showed that the proportion of CD3^+^CD4^+^T cells in the Lab/PRRSV group was significantly higher than in the Mock group at 5 dpi (*p* < 0.001). The proportion of CD3^+^CD8^+^T cells in the Lab/PRRSV group was extremely significantly higher than in the Mock group (*p* < 0.001) (Figure 3r). The proportion of CD3^+^CD4^+^T cells in the Lab/PRRSV group was significantly higher than in the Mock group at 10 dpi (*p* < 0.05). The proportion of CD3^+^CD8^+^T cells in the Mock group was significantly lower than in the Lab/PRRSV group (*p* < 0.001), while the proportion of CD3^+^CD8^+^T cells in the Lab/PRRSV group was significantly higher than in the Mock group (Figure 3s).

### 2.11. Analysis of Comparative Results of Intestinal Flora

**Figure 5:**
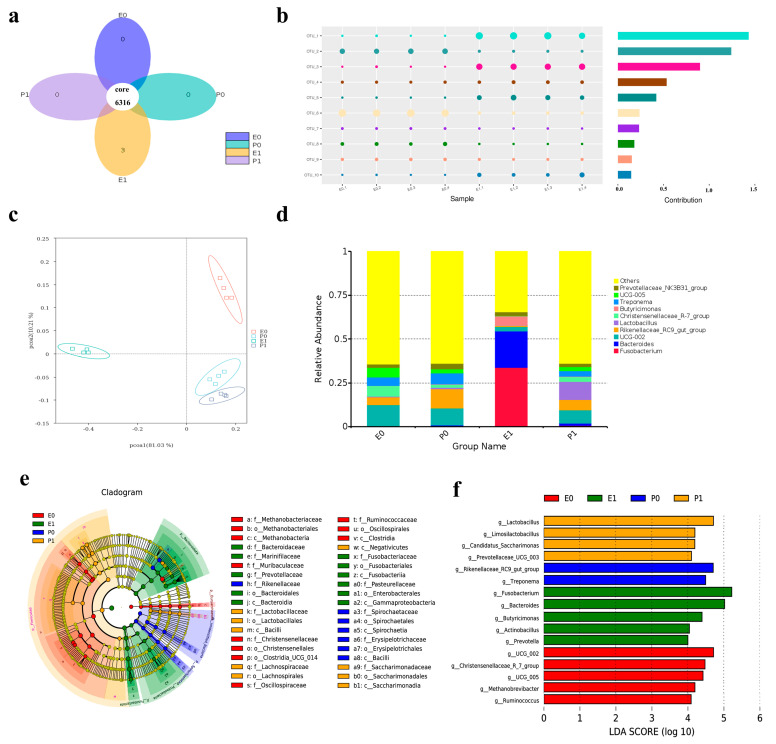
Results of small intestinal flora of piglets in each group: (**a**) the petal diagram shows the species diversity in the intestinal tract of the experimental group compared with the control group; (**b**) the bubble map shows the abundance of OTU in the intestines of the experimental group compared with the control group; (**c**) PCoA showing a comparison of species abundance in the intestinal tracts of piglets in the experimental group and the control group; (**d**) the stack histogram shows the species expression trend of intestinal flora of piglets in the experimental group compared with the control group; (**e**) LEfSe shows the different species identified in the experimental and control groups; (**f**) the histogram of LEfSe distribution shows that there are significant differences between the experimental group and the control group whose LDA score is greater than the present value. (E0 is the sample of piglets in the experimental group before challenge, and E1 is the sample of piglets in the experimental group after challenge. P0 is the experimental sample of piglets in the PBS group before challenge. P1 is the experimental sample of piglets in the PBS group after virus challenge).

**Figure 6:**
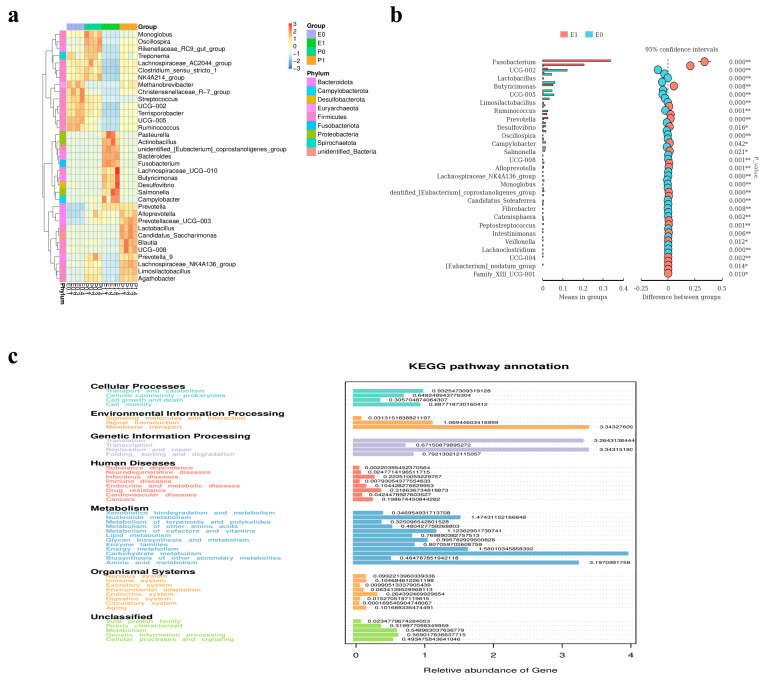
Comparison of lung transcriptome results: (**a**) the heatmap shows species diversity in the experimental and control groups at gate level; (**b**) indicator analysis showing biomarkers of species association between the experimental and control groups, (*, *p* < 0.05; **, *p* < 0.01); (**c**) KEGG enriched potential signaling pathways and biological functions in different species.

The composition and changes in the fecal microbiota of the piglets in the Lab/PRRSV group (E group) and Mock group (P group) before and after the challenge were detected. Firstly, the diversity of species composition in the feces of the piglets in the Lab/PRRSV group and Mock group before and after the challenge was compared, and it was found that the species of piglets in the Lab/PRRSV group increased after the challenge (Figure 5a,b). The results of α diversity showed that the structure of the microflora of the piglets in each group was relatively independent before and after the challenge, without intersecting sets, and the composition of the microflora species among the sequenced samples was relatively reasonable (Figure 5c). After the toxicity challenge, the relative abundance of Fusobacterium, Bacteroides, and Butyricimonas in the stool of the piglets in the Lab/PRRSV group increased significantly (Figure 5d). An LDA effect size analysis showed that a total of 38 marker species were significantly enriched in the piglet community samples of the Lab/PRRSV group and Mock group (Figure 5e). From the histogram analysis of LDA value distribution of significantly different species, it can be seen that when LDA ≥ 4, after the challenge, Fusobacterium and Bacteroides became the dominant bacteria in the gut of the piglets in the Lab/PRRSV group (Figure 5f). At the phylum level, the analysis of the difference between all samples showed that Pasteurella, Actinpbacillus, Bacteroides, Fusobacterium, and Lachnospiraceae were significantly prominent (Figure 6a). The comparison of fecal microflora at the genus level of the piglets in the Lab/PRRSV group before and after the challenge showed that the difference in Fusobacterium was significantly increased after the challenge (Figure 6b). Therefore, the KEGG database was used to analyze the pathways potentially affecting the function, and the differences in membrane transportation in the environmental information processing function were found. There were differences in translation, replication, and repair in the genetic information processing function. There was a difference in carbohydrate metabolism in the metabolic function (Figure 6c).

### 2.12. Analysis of Transcriptome Comparison Results

**Figure 7:**
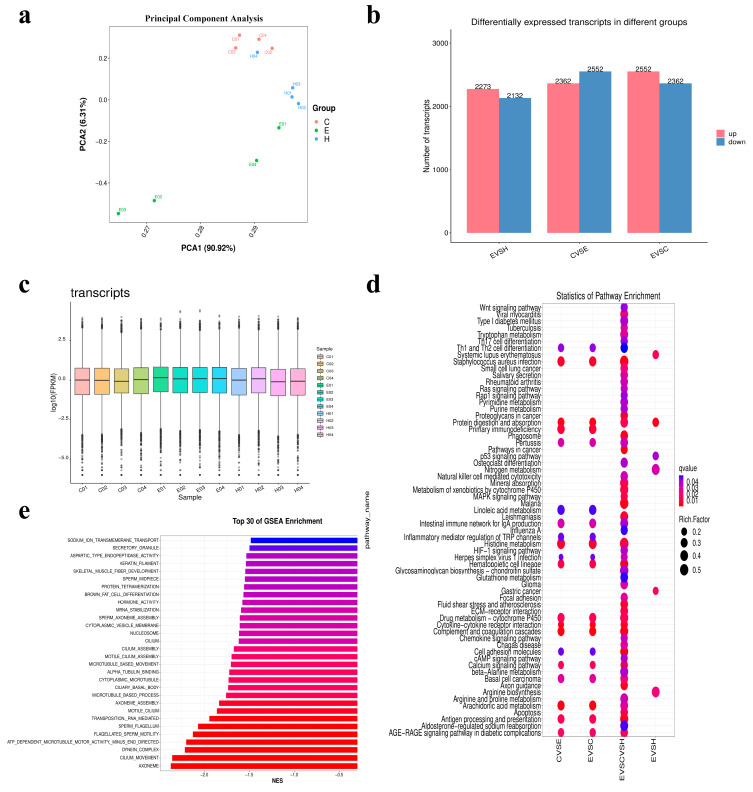
Lung transcriptome differential genes and differential pathway results: (**a**) comparison of pulmonary samples in experimental group and control group using principal component analysis; (**b**) the down-regulation of differential genes among different groups was compared; (**c**) the number of transcribed products in the lungs of different groups was compared; (**d**) differential pathway enrichment statistics caused by differential genes; (**e**) the top 30 differential signaling pathways in the GSEA-Go database were enriched.

To understand the function of probiotics in the process of PRRSV infection in piglets, the lung tissues of the Lab/PRRSV group (E1–E4), Mock group (C1–C4), and healthy group (H1–H4) were sequenced with transcriptome technology. The PCoA method was used to analyze the differences in community structure among the three groups of piglet lung samples, and the results showed differences in the distribution of samples among the Lab/PRRSV (E1–E4), Mock (C1–C4), and healthy piglet (H1–H4) groups (Figure 7a). The differentially expressed transcribed sample genes between the different groups were compared and statistically analyzed. The results showed that 2362 differentially expressed genes were up-regulated and 2552 differentially expressed genes were down-regulated in the Mock group compared with the Lab/PRRSV group (Figure 7b). The reading values of each transcript sample in the Lab/PRRSV group were significantly higher than those in the Mock group (Figure 7c). Through a comparative analysis of the lung differential gene enrichment and KEGG pathway library of the three groups, significant differences were found in the Th1 and Th2 cell differentiation pathway, influenza infection pathway, glutathione metabolism pathway, and aldosterone regulation sodium reabsorption pathway, which may indicate that the piglets were infected with PRRSV after immune probiotics were administered. They may regulate cell differentiation, immune function, and viral infection in piglets (Figure 7d). GSEA made it possible to include differential pathways, where more subtle and coordinated changes affect biological pathways, so the top 30 differential pathways were enriched, and the results showed that sodium ion transmembrane transportation, secretory granule, aspartic type endopeptidase activity, and other biological functions were affected (Figure 7e). These results suggested that PRRSV infection may affect the health of pigs by affecting the biological functions of sodium transport and aspartate endopeptidase activity in the lungs.

## 3. Discussion

*Lactobacillus plantarum*, as the dominant species of lactic acid bacteria, is a highly effective delivery vector for expressing foreign proteins [13]. It has strong adhesion, hydrophobicity, and no pathogenic properties [14]. Immunization through oral or nasal routes can colonize the mucosal site, stimulate mucosal immunity, strengthen the mucosal barrier, and play an important role in the protection and immune regulation of the body and in the health of humans and animals [15]. It has certain application prospects. In addition, the greatest advantage of L. plantarum is that it can induce intestinal mucosal immune function to produce sIgA, which can be used to resist pathogen infections [16]. PRRSV is an immunosuppressive disease that seriously affects the host’s immune system [17]. At present, most vaccines are designed to induce immune responses in the body to protect the host, but their safety is still a difficulty to overcome [18]. Mucosal immunity may be the most effective method to suppress PRRSV infection [19]. In this study, the mucosal immunization method was used to block PRRSV infection to protect the health of piglets, and clinical trials were conducted to understand the protective mechanism.

Based on the sequence of the mAb-PN9cx3 antibody, novel functional *Lactobacillus plantarum* expressing pn9CX3-scFv, pn9CX3-HC, and pn9CX3-LC was constructed using homologous recombination and molecular cloning techniques. The proteins of PN9cx3-scFV and PN9cx3-HC expressed in prokaryotes inhibited PRRSV in vitro. Lv, J., et al. optimized the expression of a PRRSV ORF5 protein and PCV2 ORF2 protein in Lactococcus lactis [20]. Wayah, S.B et al. cloned the PRRSV GP5 gene into lactic acid bacteria [21]. Tanweer, F et al. used code-optimized truncated GP5 gene 5′ and 3′ ends linked to endogenous signal peptide (DCpep) and target peptide (Mpep), respectively, and displayed them on the surface of *Lactobacillus plantarum*, showing high antigenicity [22]. In animal experiments, the piglets in the Mock group showed symptoms of drowsiness, sneezing, and swollen eyelids [23], and their body temperature was higher than those in the Lab group and Mock group. At the same time, their body weight decreased over time, while in the Lab group, the virus was infecting the piglets in the Mock group, affecting their health. However, the piglets in the Lab group were similar to those in the Mock group, and the virus had no obvious effect on the piglets.

The viral load in all organs of the Mock group was significantly higher than in the Lab group (Lab/PRRSV), and the differences in pulmonary and hilar lymph nodes were extremely significant (*p* < 0.01). At this time, the virus was replicated in the organs of piglets in each group, but the piglets treated with oral *Lactobacillus plantarum* had lower viral loads. On the 5th and 10th days after the challenge, the viral load in the serum of the piglets in the Mock group was significantly higher than in the piglets in the Lab group, and the body temperature was also increasing. The virus was released into the blood after replication from the organs, and viremia occurred [24]. Based on the immunofluorescence, a large number of virus particles were observed in the tissue, and in the pathological examination, the tissue was damaged by the virus [25]. The piglets fed with compound lactic acid bacteria were less damaged by the virus, which was similar to the piglets in the healthy group [26].

T lymphocytes play an important role in the prevention of PRRSV infection; in particular, CD3^+^CD8^+^T lymphocytes have the role of PRRSV clearance to protect the body from the virus [27]. The compound *Lactobacillus plantarum* can significantly increase the proportion of CD3^+^CD8^+^T cells in the peripheral blood of pigs, and these cells help the body to resist PRRSV infection and play a positive role in the immune system. The complex *Lactobacillus plantarum* can significantly inhibit the depletion of T lymphocytes caused by the virus and plays a positive role in the clearance process of PRRSV. Wang Jianzhong et al. constructed recombinant Lactococcus lactis expressing the PRRSV ORF6 gene, and their results showed that mice immunized using nasal drops could enhance the Th1 mucosal immune response, enhance the secretion of sIgA, and significantly enhance the secretion levels of cytokines such as IL-2 and IFN-γ [28]. Th1 cells mainly secrete interleukin-2 (IL-2), interferon-γ (IFN-γ), and tumor necrosis factor-β (TNF-β). These cytokines play a key role in cellular immunity. IFN-γ can activate macrophages, enhance their phagocytic and bactericidal ability, and induce the expression of MHC class I and class II molecules, which enhance antigen presentation. The cytokines secreted by Th2 cells mainly include interleukin-4 (IL-4), IL-5, IL-10 and IL-13. They play a dominant role in the humoral immune response. For example, IL-4 can promote the proliferation and differentiation of B cells and induce IgE antibody production by B cells. IL-10 has an immunosuppressive function, can inhibit the activity of Th1 cells and macrophages, and regulates the balance of immune response. In this study, the changes in IL-10, IL-6, TNF-α, and IFN-γ in the serum of piglets in each group were detected, and the levels of IL-10 and IFN-γ were significantly higher in piglets immunized with Lactobacillus complex after PRRSV infection than those in the PBS group. The content of IL-6 and TNF-α was significantly lower than that of the PBS group, and Lactobacillus affected the T cell immune response of piglets.

Gut microbiota plays a crucial role in regulating the metabolism of the host, keeping it steady [29]. Virus infection directly affects the immune system and health of piglets, and when the immune system is damaged, it indirectly affects the changes in their intestinal microflora [30]. On the sixth day after piglets were infected with PRRSV, severe diarrhea occurred. At this time, the intestinal microflora environment of the piglets was damaged, and opportunistic pathogens increased, which seriously affected the absorption of nutrients in food. The relative abundance of Fusobacterium, Bacteroides, and Butyricimonas in the feces of piglets in the Lab/PRRSV group increased significantly after the challenge, which may be related to the change in the species diversity of the intestinal flora of piglets after feeding them complex Lactobacillus. In the face of virus infection, more beneficial bacteria were activated to protect the intestinal flora balance of the piglets, and the number of lactic acid bacteria was also significantly increased. Membrane transportation affects the environmental information processing function, while translation, replication, and repair affect the genetic information processing function, which also indicates the importance of this bacterial group in resisting virus infections [31]. Recombinant lactic acid bacteria have been exposed to artificial intestinal fluid, artificial gastric fluid, and bile and can survive easily. During the construction of recombinant lactic acid bacteria, we added a variety of adhesion factors; for example, the surface proteins of lactic acid bacteria (such as S-lamin) can bind to the glycoproteins or glycolipids on the surface of intestinal epithelial cells. Lactic acid bacteria can also enhance their adhesion ability by secreting exopolysaccharides, which can interact with components in the mucous layer of the gut to help the Lactobacillus secure its position in this layer; once adhesion is successful, the recombinant Lactobacillus begins to colonize the gut. The piglets use the nutrients in their gut to grow and reproduce. During colonization, lactic acid bacteria also interact with each other. They may form biofilms, which can provide a relatively stable living environment for lactic acid bacteria.

Transcriptome technology can reveal the underlying gene expression differences in animals under the influence of different treatments [32]. After PRRSV infection, 2362 differential genes were up-regulated, and 2552 differential genes were down-regulated in piglets fed composite Lactobacillus. Compared with the database after the enrichment of differential genes, the Th1 and Th2 cell differentiation pathway, influenza infection pathway, glutathione metabolism pathway, and aldosterone regulation of sodium reabsorption were affected by the virus, which may suggest that the regulation of cell differentiation, immune function, and the virus infection process were affected after piglets were infected with PRRSV by immune probiotics. Through GSEA, the differential pathways with more subtle and coordinated changes affecting biological pathways could be included, and the top 30 differential pathways were enriched [33]. Sodium ion transmembrane transportation, secretory granule, aspartic type endopeptidase activity, and other biological functions were affected. These results suggest that PRRSV infection may affect the health of pigs by affecting the biological functions of sodium ion transportation and aspartate endopeptidase activity in the lungs [34].

The Lactobacillus expression vector mentioned in this article has limitations, which limit its ability to express foreign proteins. Our next study will address this issue. Through the optimization of the vector sequence and components, the protein expression of Lactobacillus can be improved to achieve a better immune protection effect.

## 4. Materials and Methods

### 4.1. Strains and Preservation

The recombinant *Lactobacillus plantarum* used in this study (for delivering single-chain antibody proteins) is preserved by the Research and Development Center for Animal Microecological Preparations at Jilin Agricultural University, PRC. All recombinant *Lactobacillus plantarum* samples were cultured at 37 °C in an MRS Medium. The pSIP409-pgsA vector plasmid was stored in *Escherichia coli* 6212, and after transformation into the *Lactobacillus plantarum* strain NC8 (CCUG 61730), 10 μg/mL of erythromycin was added. The mAb-PN9cx3 gene sequence was synthesized by Professor Nan Yuchen from Northwest A&F University, and three groups of target genes, PN9cx3-scFV, PN9cx3-HC, and PN9cx3-LC, were synthesized into a pET28a vector by Jinsirui Biotechnology Co., Ltd., Jiangsu, China. The virus PRRSV-JXA1 was donated by Professor Jin Ningyi’s team from the Institute of Military Veterinary Medicine, Academy of Military Medical Sciences (Changchun, China) [35].

### 4.2. Construction of Recombinant Lactobacillus plantarum

To construct *Lactobacillus plantarum* anchored to express the recombinant small molecular antibodies PN9cx3-scFV, PN9cx3-HC, and PN9cx3-LC, three groups of target genes, i.e., PN9cx3-scFV, PN9cx3-HC, and PN9cx3-LC, were synthesized by Genzyme Biotechnology Co., Ltd., Nan jing, China. The vector pSIP409-pgsA’(E) and the target gene were connected using the seamLess cloning method and transformed into *Escherichia coli* 6212 competent cells. The plasmid was extracted and transformed into *Lactobacillus plantarum* NC8 using electroporation. The resulting strains were named NC8/pSIP409-pgsA ‘-PN9cx3-scFV(E), NC8/pSIP409-pgsA’ -PN9cx3-HC(E), and NC8/pSIP409-pgsA ‘-PN9cx3-LC(E).

### 4.3. Western Blot

The three successfully constructed recombinant L. plantarum strains were activated and inoculated in a 5 mL MRS liquid medium and incubated overnight at 37 °C in an anaerobic workstation. The next day, the bacteria were transferred to a 50 mL centrifuge tube, and all recombinant *Lactobacillus plantarum* samples were cultured at 37 °C under anaerobic conditions. When the OD 600 value reached 0.3, SPPIP-inducing peptide (50 ng/mL) was added. After induction, the recombinant *Lactobacillus plantarum* was collected and repeatedly freeze–thawed at −80 °C 5 times, after which the protein samples were processed. Subsequently, SDS-PAGE was performed on a 10% acrylamide gel, and after being transferred to the membrane, the membrane was blocked with 5% skimmed milk powder for 1 h at room temperature. A 1:5000 dilution of murine His-tag mab was incubated as a primary antibody in a shaker overnight at 4 °C and washed five times using TBST. Finally, an ECL chromogenic kit was used for color development.

### 4.4. PRRSV Titer Determination

MARC-145 cells were spread in a 96-well cell culture plate at a cell volume of 2.5 × 10^4^ per well, and the length of the paste was 90%. The virus was diluted with DMEM (high glucose), and 100 μL/well of 7 different concentrations of virus dilutions from 10^−1^ to 10^−7^ was added to the cells in the 96-well plate. Eight replicates were made for each concentration group. An equal volume of DMEM (high glucose) containing a 1% double antibody was added to the cells as the control group, and an equal volume of virus stock solution was added to the cells as the positive control group. The cells were placed in a cell incubator at 37 °C, and the cytopathic effect was carefully observed at all times within 3–7 days. TCID50 per 0.1 mL was calculated according to the formula log_10_TCID50 = L − D (S − 0.5), where L is the log of the highest dilution, D is the difference between the log of dilutions, and S is the sum of the positive well ratios [36].

### 4.5. Cytotoxicity Test

The purified small-molecule proteins of three single-chain antibodies were filtered with a 0.22 μm filter and diluted with DMEM (high sugar) in ratios of 1:2, 1:4, 1:8, 1:16, 1:32, 1:64, 1:128, and 1:266. MARC-145 cells were cultured in the 96-well plate to 80% cells, 50 μL diluted protein was added to each well, 50 μL medium was also added to each well, and the cells were incubated in a cell incubator at 37 °C for 72 h. Next, 10 μL of a CCK-8 detection reagent was added to each well; the cells were incubated at 37 °C for about 3 h in the dark, and OD450nm-reading was performed. The effects of PN9cx3-scFV, PN9cx3-HC, and PN9cx3-LC on cell viability were detected [37].

### 4.6. Three Groups of Proteins Against PRRSV Effect Research

The purified small-molecule protein was diluted in DMEM (high glucose) in ratios of 1:2, 1:4, 1:8, 1:16, 1:32, 1:64, 1:128, and 1:256. The PRRSV solution was diluted to 200TCID50/50 μL. The protein concentration was 50 μL/well. Virus diluent and 50 μL/porin diluent were mixed into MARC-145 cells in a 96-well plate, incubated at 37 °C for 1 h, and DMEM (H) containing 4% FBS and 1% double antibody was added at 100 μL/well. Negative control and positive control holes were set up and incubated at 37 °C for 72 h, and the cell supernatant was collected and stored in a refrigerator at −80 °C [38].

### 4.7. Quantification of Virions in Cells

The viral nucleic acid was extracted from MARC-145 cells and reverse-transcribed into cDNA. The viral particles were quantitatively analyzed using qRT-PCR [39]. First, plasmid standards were constructed based on PRRSV-JXA1(GenBank): the conserved region gene sequence of EF112445.1 was synthesized using bioengineering technology, and the target gene of JXA1(13578–13717) with a size of 140 bp, which was cloned into the pUC-GW-Kan vector and used as the standard plasmid for absolute quantization of qRT-PCR, and the qRT-PCR primers were designed and synthesized. Primer names: the sequence of primers was JXA1-F: AACGCTCCTTAGTGGTCGATC, JXA1-R GTCAAGCACTTCCCCAACATAC. Then, the concentration of standard plasmid pUC-GW-JXA1-Kan was diluted to 10^−1^, 10^−2^, 10^−3^, 10^−4^, and 10^−5^ ng/μL to establish the standard curve, and the copy number was calculated using the following formula: Copies/(including L = (6.02 × 10^23^ copy number/Moore) × (ng/(including L × 10^−9^)/(DNA/bp × 660) [40]. The reaction procedure was as follows: the predenaturation temperature was 95 °C for 3 min, and after cyclic amplification, the denaturation was 95 °C for 20 s, and the annealing temperature was 55 °C for 20 s. The extension temperature was 72 °C for 30 s, for a total of 30 cycles.

### 4.8. Design and Grouping of Animal Experiments

In this experiment, 30-day-old landrace piglets were used and divided into three groups, i.e., a Mock/PRRSV group (*n* = 4), Mock group (*n* = 4), and Lab (LP)/PRRSV group (*n* = 4), in which the Mock/PRRSV group was the healthy control group, and the Mock group was administered with PBS after injection. Piglets in the Lab/PRRSV group were given complex recombinant Lactobacillus orally (3 × 10^10^ CFU per pig). Each strain was 1 × 10^10^ CFU/mL). On the 14th day, piglets were challenged with a nasal spray, and the dosage of the challenge was 200TCID50/1 mL. The condition of the piglets in each group was observed, and their body temperature and body weight were measured [41].

### 4.9. Flow Cytometry

Blood samples of 5 mL were collected from the anterior vena cava on days 7 and 14 of feeding with compound *Lactobacillus plantarum* and on days 5 and 10 of the challenge. The blood was lysed with red blood cell lysate for 10 min, centrifuged at 4 °C for 5 min, and then the supernatant was discarded, and cell counts were performed under a microscope. The cell suspension (2 × 10^6^ cells) was aspirated for later use. Anti-porcine CD3-PE-Cy7 (BD Company, Franklin Lakes, NJ, USA), anti-porcine CD4-PE (BD Company), and anti-porcine CD8-FITC (BD Company) were diluted at a ratio of 1:4, and 10 μL of an anti-porcine CD3 antibody, 10 μL of an anti-porcine CD4 antibody, and 10 μL of an anti-porcine CD8 antibody were added to each 2 × 10^6^ cell suspension. Incubation occurred at 4 °C for 20 min; then, they were centrifuged at 400× *g* for 5 min at 4 °C. Cell precipitations were gently blown with 1 mL FACS and centrifuged at 4 °C for 5 min. This step was repeated twice, and the cells were re-suspended at 300 μL FACS and examined on an LSRfortessa™ cell analyzer (BD Biosciences, Franklin Lakes, New Jersey, USA). All data were analyzed using FlowJo7.6.2 software.

### 4.10. Pathological Anatomical Observation

The piglets were euthanized, and each piglet was injected with 2 mmol/kg potassium chloride solution intravenously. The pathological changes in the heart, liver, spleen, lungs, kidneys, and other organs of the piglets were carefully observed and recorded during slaughter and sampling. The piglet carcasses were treated harmlessly.

### 4.11. Hematoxylin–Eosin Staining

Appropriately sized tissues and organs were cut and fixed in 4% paraformaldehyde for 5–7 days. After the completion of fixation, the tissues were removed and placed into the tissue embedding box, rinsed continuously with running water for 10–12 h, followed by dehydration, transparency, wax infiltration, sectioning, baking, and other procedures, and finally stained with hematoxylin dye and observed with a microscope.

### 4.12. Enzyme-Linked Immunosorbent Assay

The serum in the blood of piglets in each group was collected, and the changes in IL-6, IL-10, TNF-α, and IFN-γ in the serum were detected using an ELISA kit (Jiangsu Enzyme Free Biology Co., Ltd., Jiangsu, China). The inflammatory response of piglets in each group was evaluated after PRRSV infection.

### 4.13. Indirect Immunofluorescence

The tissues and organs fixed on slides were incubated with PBS containing 10% pig serum for 30 min. The PRRSV serum and Fluor 488-labeled goat anti-pig IgG(H+L) (Biyantian Biotechnology Co., Ltd., Jiangsu, China) were mixed and added to the slides overnight at 4 °C. Subsequently, the slides were washed three times with PBS for 5 min each, and the nuclei were stained with a 4, 6-diamino-2-phenylindole (DAPI) solution (Invitrogen, New York, NY, USA) for 5 min. After that, the slides were washed three times with PBS for 5 min each. The samples were sealed using an anti-quench seal. The slides were imaged using a DMi8 fluorescence inverted microscope (Leica Instruments GMBH, Berlin, Germany).

### 4.14. Quantification of Virus Particles in Various Tissues and Organs

The viral nucleic acids were extracted from the lungs, hilar lymph node, spleen, heart, kidneys, mesenteric lymph node, duodenum, jejunum, and ileum of piglets in each group after the challenge, and the viral particles were quantitatively analyzed via qRT-PCR.

### 4.15. Intestinal Microbiota Sequencing

Fresh feces of piglets in each group were collected, labeled, and quickly frozen in liquid nitrogen. The samples were sent to Beijing Nohe Zhiyuan Biological Co., Ltd., Beijing, China, for sequencing. First, the DNA of each group’s feces was extracted. 341F(5′-CCTAYGGGRBGCASCAG-3′) and 806R(5′ggactacnngggtatctaat-3′) priors were used to amplify the V3–V4 hypervariable region of the intestinal bacteria 16S rRNA gene. An Illumina NovaSeq sequencing platform was selected for analysis, a small fragment library was constructed based on the Illumina NovaSeq sequencing platform, and sequencing was performed through Paired_End. After data quality control, data reading splicing and filtering, operational taxon (OTU) clustering, species annotation and abundance analysis, and in-depth data mining based on α diversity and β diversity and functional prediction data were performed to interpret the intestinal flora sequencing results. The relationship between intestinal flora and disease was analyzed, and the mechanism of intestinal flora in health and disease was discussed [42].

### 4.16. Transcriptome Sequencing

Lung tissues of the same location and size were obtained from piglets in each group on a low-temperature operating table. The samples were placed in the freeze-storage tube, marked, and quick-frozen with liquid nitrogen and sent to Beijing Nohe Technology Co., Ltd. for transcriptome sequencing. The TRIzol method was used to extract the total RNA from the lungs, and RNA quality control was carried out [43]. A reverse transcription kit and library construction kit were used to construct RNA libraries. The library was quantified to detect its quantity and ensure its quality. The library was sequenced with Illumina using paired-end double-ended sequencing, with data including read1 and read2 pairs. The Pearson correlation coefficient between the samples and principal component analysis (PCA) can be used to understand the repeatability of samples and help exclude outliers [44]. Differentially expressed genes refer to the summary of up-regulated and down-regulated genes between samples or in the same sample after different treatments, and genes were usually screened from the two aspects of difference multiple and significance levels. The input data for differential expression analysis of genes (transcripts) were the data of reads [45]. The *p*-value calculation model based on a negative binomial distribution was used for *p*-value calculation, and BH was used for *p*-value correction to obtain the q-value. The difference multiple was the difference between the mean expression of the experimental group and the mean expression of the control group. Cluster heatmap was used to display differential gene expression patterns, and GO and KEGG pathway enrichment analysis was performed on differentially expressed genes. Gene enrichment analysis refers to the process of classifying genes according to genome annotation information or database annotation information. After the classification of genes, it can help us to recognize whether the genes found have certain aspects in common (function, pathway, etc.). Through enrichment analysis of differentially expressed genes, it is expected to find pathways that play key roles in biological processes so as to explore the basic molecular mechanisms that reveal biological processes [46].

### 4.17. Statistical Analysis

Flow cytometry was performed using FlowJo7.6.2 software. GraphPadPrism software 9.0 was used to make charts and conduct statistical analysis. A one-way analysis of variance was used for differences between groups (*, *p* < 0.05; **, *p* < 0.01; ***, *p* < 0.001; ****, *p* < 0.0001). The data maps of intestinal flora and transcriptome in this study were produced by Beijing Nohe Zhiyuan Biological Co., Ltd.

## 5. Conclusions

The novel functional Lactobacillus expressing PRRSV antigen has an inhibitory effect on PRRSV infection. This study provides a feasible scheme for further understanding PRRSV infections and the mechanism of probiotics.

## Data Availability

The data generated in this study and an analysis of the data are included in this article (and its supplementary information file) for publication. The flora data have been uploaded to the GSA (Genome Sequence Archive) database under the upload number PRJCA010756, and the transcriptome sequencing data have been uploaded to the database under the upload number PRJCA011370. The PRRSV used in this article was donated by the Institute of Military Veterinary Medicine, Medical College of Military Science, Chinese Academy of Sciences. The new functional lactic acid bacteria constructed in this study were stored at the College of Animal Medicine, Jilin Agricultural University.

## Supplementary Materials

The following supporting information can be downloaded at https://www.mdpi.com/article/10.3390/ijms26052257/s1.
