# Supplementary material for: Lactobacillus plantae Expressing Porcine Reproductive and Respiratory Syndrome Virus (PRRSV) Single-Chain Antibody Can Inhibit PRRSV Replication and Change the Intestinal Flora Structure of Piglets
Source: Int J Mol Sci. 2025 Mar 3;26(5):2257. doi: 10.3390/ijms26052257 (PMC11901011; doi:10.3390/ijms26052257)
Supplement: Supplementary file 1 [file ijms-26-02257-s001.zip › ijms-3450012-supplementary.pdf]

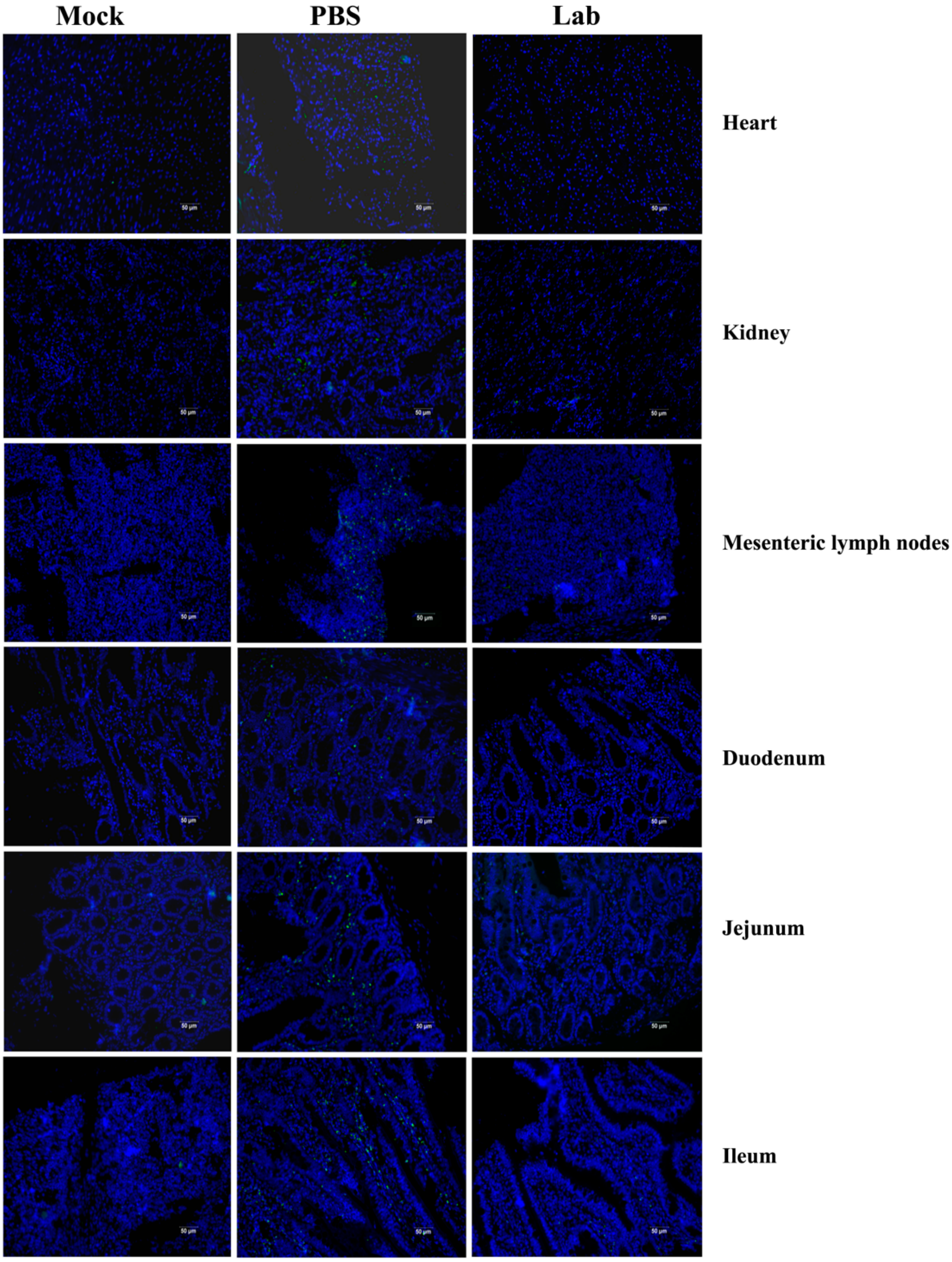

Figure S1. Immunofluorescence supplementary image of virus in tissue.

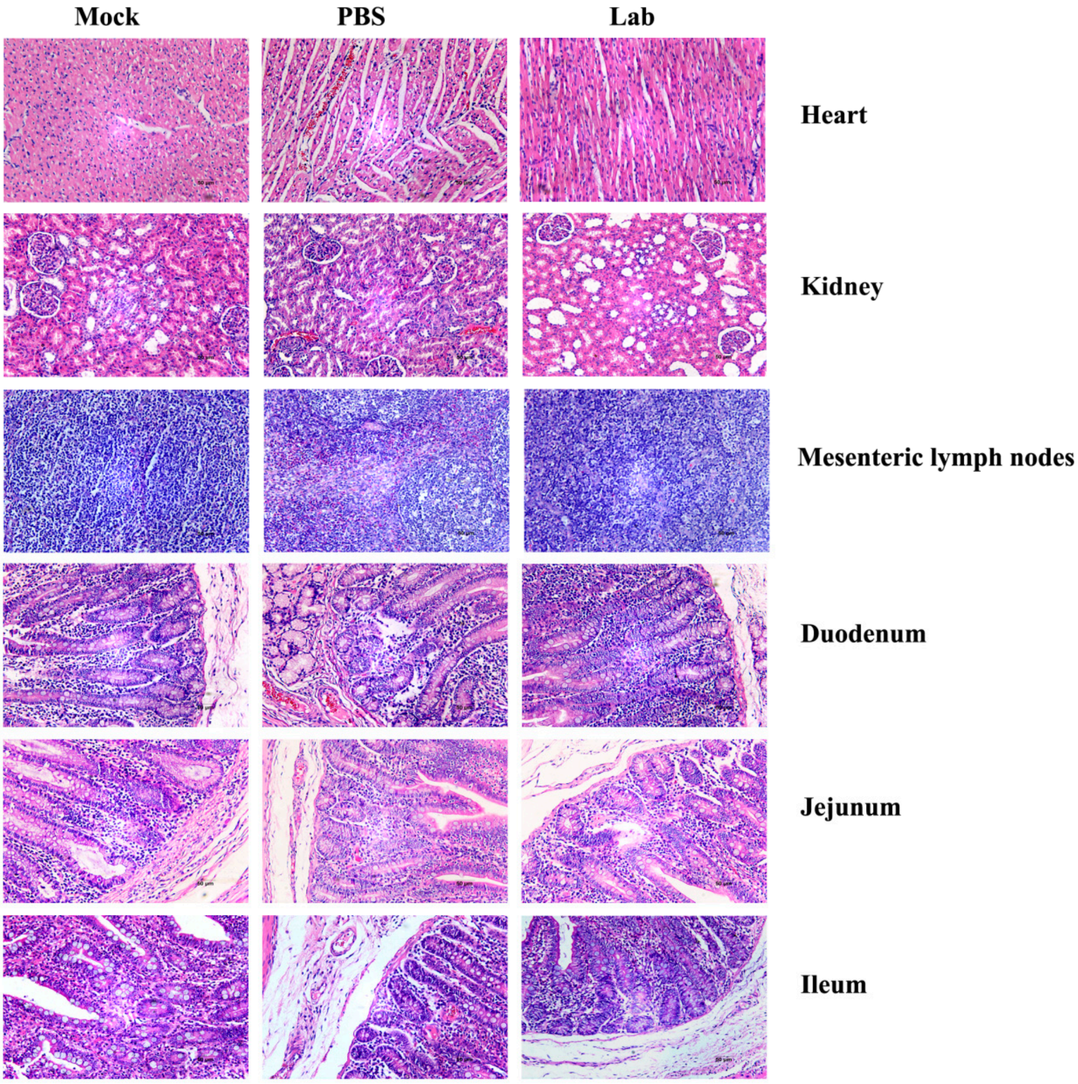

Figure S2. Supplementary histological section of tissue.
